# Supplementary material for: Odor descriptive ratings can predict some odor-color associations in different color features of hue or lightness
Source: PeerJ. 2023 Apr 20;11:e15251. doi: 10.7717/peerj.15251 (PMC10122842; doi:10.7717/peerj.15251)
Supplement: Supplemental Information 1 [file peerj-11-15251-s001.docx]

## Supplementary method 1: Sex differences of the odor-color responses.

We used Bayesian multilevel regression models to estimate the sex differences of color responses using odor-level effects. The response value of a*-axis for the associated colors was modeled by each odor as a normal distribution as follows:

$$a_{m\left[ j \right]}^{*}\sim Normal\left( \alpha_{m\left[ i \right]}, \sigma_{ma\left[ i \right]} \right)$$

$\sigma_{ma[i]}$ > 0

$$a_{f[j]}^{*}\sim Normal(\alpha_{f[i]}, \sigma_{fa[i]})$$

$\sigma_{fa[i]}$ > 0

where *i* indicates the odor ID and *j* denotes the data index. $a_{m\left[ j \right]}^{*}$ indicates the responses from male participants, and $a_{f\left[ j \right]}^{*}$ were from female participants. Each coefficient followed a normal distribution with mean coefficients as follows:

$$\alpha_{m[i]} \sim Normal(\alpha_{m0} , \sigma_{ma0})$$

$$\alpha_{f[i]} \sim Normal(\alpha_{f0} , \sigma_{fa0})$$

$\sigma_{ma0}$ > 0

$\sigma_{fa0}$ > 0

where $\alpha_{m0}$ and $\alpha_{f0}$ indicates the average coefficients across all odorants in male and female participants.

The differences between sex were calculated from the estimated parameters as follows:

$${\Delta\alpha}_{mf0}= \alpha_{m0}- \alpha_{f0}$$

$${\Delta\alpha}_{mf[i]}= \alpha_{m[i]}- \alpha_{f[i]}$$

with ${\Delta\alpha}_{mf[i]}$ indicating the coefficient differences in *i*th odor, and ${\Delta\alpha}_{mf0}$ indicating the main effect of sex across all odors.

Regarding the model for a* value estimation, we modeled the b*- axis values as follows:

$$b_{m\left[ j \right]}^{*}\sim Normal\left( \beta_{m\left[ i \right]}, \sigma_{mb\left[ i \right]} \right)$$

$\sigma_{mb[i]}$ > 0

$$b_{f[j]}^{*}\sim Normal(\beta_{f[i]}, \sigma_{fb[i]})$$

$\sigma_{fb[i]}$ > 0

$$\beta_{m[i]} \sim Normal(\beta_{m0} , \sigma_{mb0})$$

$$\beta_{f[i]} \sim Normal(\beta_{f0} , \sigma_{fb0})$$

$\sigma_{mb0}$ > 0

$\sigma_{fb0}$ > 0

$${\Delta\beta}_{mf0}= \beta_{m0}- \beta_{f0}$$

$${\Delta\beta}_{mf[i]}= \beta_{m[i]}- \beta_{f[i]}$$

Similar to the steps with a* and b*, we model the L-axis values as follows.

$$L_{m\left[ j \right]}^{*}\sim Normal\left( \lambda_{m\left[ i \right]}, \sigma_{mL\left[ i \right]} \right)$$

$\sigma_{mL[i]}$ > 0

$$L_{f[j]}^{*}\sim Normal(\lambda_{f[i]}, \sigma_{fL[i]})$$

$\sigma_{fL[i]}$ > 0

$$\lambda_{m[i]} \sim Normal(\lambda_{m0} , \sigma_{mL0})$$

$$\lambda_{f[i]} \sim Normal(\lambda_{f0} , \sigma_{fL0})$$

$\sigma_{mL0}$ > 0

$\sigma_{fL0}$ > 0

$${\Delta\lambda}_{mf0}= \lambda_{m0}- \lambda_{f0}$$

$${\Delta\lambda}_{mf[i]}= \lambda_{m[i]}- \lambda_{f[i]}$$

The models were fitted using the R environment (ver.3.4.0) and RStan (ver.2.2.1) with the Markov chain Monte Carlo (MCMC) method. All estimates were made with 3,000 samplings, running four chains to generate random numbers, and a burn-in period of 1,000. We used the Gelman-Rubin statistics $\hat{R}$ to determine if the MCMC estimation converged for all estimation parameters. $\hat{R}$ is generally considered to converge as it approaches 1.10, and each model fit produces $\hat{R}$ <1.10.

The mean differences and their 95% CI of main effects and odor-level differences were estimated using these multilevel models. The posterior distribution and its 95% CI for sex differences did not include 0. These results indicated that the color responses were not significantly fluctuated between sex groups in response to any odorant.
