# Supplementary material for: Odor descriptive ratings can predict some odor-color associations in different color features of hue or lightness
Source: PeerJ. 2023 Apr 20;11:e15251. doi: 10.7717/peerj.15251 (PMC10122842; doi:10.7717/peerj.15251)
Supplement: Supplemental Information 2 [file peerj-11-15251-s002.docx]

## Supplementary method 2: Multilevel regression analysis including intercepts from individual errors.

We modeled the response value of the a*-axis for the associated colors by each odor as a normal distribution. The model included intercepts $\tau_{a[k]}$ of individual errors as follows:

$$a_{[j]}^{*}\sim Normal(\alpha_{0\left[ i \right]}+\alpha_{S\left[ i \right]}{Strength}_{\left[ i \right]}+ \alpha_{P\left[ i \right]}{Pleasantness}_{\left[ i \right]}+ \alpha_{F\left[ i \right]}{Familiarity}_{\left[ i \right]}+ \alpha_{E\left[ i \right]}{Edibility}_{\left[ i \right]}+ \alpha_{A\left[ i \right]}{Arousal}_{\left[ i \right]}+ \tau_{a[k]}, \sigma_{a[i]})$$

$\sigma_{a[i]}$ > 0

$\tau_{[k]}$ > 0

where *i* indicates the odor ID, *j* denotes the data index, and *k* denotes participant ID. In this model, the intercept term was $\alpha_{0[i]}$ + $\tau_{a[k]}$. The intercept $\alpha_{0[i]}$, and each coefficient, $\alpha_{X[i]}$, followed a normal distribution with mean coefficients as described in Manuscript.

The intercept $\tau_{a[k]}$ followed a normal distribution as below:

$$\tau_{a[k]} \sim Normal(0 , \tau_{a0})$$

$$\tau_{a0}>0$$

where $\tau_{a0}$ indicates the standard deviation of $\tau_{a[k]}$ among all participants.

Regarding the model for a* value estimation, we modeled the b*-axis values as follows:

$$b_{[j]}^{*}\sim Normal(\beta_{0\left[ i \right]}+\beta_{S\left[ i \right]}{Strength}_{\left[ i \right]}+ \beta_{P\left[ i \right]}{Pleasantness}_{\left[ i \right]}+ \beta_{F\left[ i \right]}{Familiarity}_{\left[ i \right]}+ \beta_{E\left[ i \right]}{Edibility}_{\left[ i \right]}+ \beta_{A\left[ i \right]}{Arousal}_{\left[ i \right]}+ \tau_{b[k]}, \sigma_{b[i]})$$

The intercept $\tau_{b[k]}$ followed a normal distribution as below:

$$\tau_{b[k]} \sim Normal(0 , \tau_{b0})$$

$$\tau_{b0}>0$$

where $\tau_{b0}$ indicates the standard deviation of $\tau_{b[k]}$ among all participants.

Similar to the steps with a* and b* values, we model the L-axis values as follows:

$$L_{[j]}^{*}\sim Normal(\lambda_{0\left[ i \right]}+\lambda_{S\left[ i \right]}{Strength}_{\left[ i \right]}+ \lambda_{P\left[ i \right]}{Pleasantness}_{\left[ i \right]}+ \lambda_{F\left[ i \right]}{Familiarity}_{\left[ i \right]}+ \lambda_{E\left[ i \right]}{Edibility}_{\left[ i \right]}+ \lambda_{A\left[ i \right]}{Arousal}_{\left[ i \right]}+ \tau_{L0} , \sigma_{L[i]})$$

The intercept $\tau_{L[k]}$ followed a normal distribution as below:

$$\tau_{L[k]} \sim Normal(0 , \tau_{L0})$$

$$\tau_{L0}>0$$

where $\tau_{L0}$ indicates the standard deviation of $\tau_{L[k]}$ among all participants.
